# Long-Term Real-World Outcomes of First-Line Pembrolizumab Monotherapy for Metastatic Non-Small Cell Lung Cancer With ≥50% Expression of Programmed Cell Death-Ligand 1

**DOI:** 10.3389/fonc.2022.834761

**Published:** 2022-03-25

**Authors:** Vamsidhar Velcheti, Xiaohan Hu, Lingfeng Yang, M. Catherine Pietanza, Thomas Burke

**Affiliations:** ^1^ Perlmutter Cancer Center, New York University, New York, NY, United States; ^2^ Center for Observational and Real World Evidence, Merck & Co., Inc., Kenilworth, NJ, United States; ^3^ Clinical Research, Merck & Co., Inc., Kenilworth, NJ, United States

**Keywords:** non-small cell lung cancer (NSCLC), manual chart review, observational study, pembrolizumab, real-world progression-free survival (rwPFS), overall survival (OS), tumor response assessment

## Abstract

**Objectives:**

Immune checkpoint inhibitors (ICIs) of programmed cell death 1/programmed cell death ligand 1 (PD-1/PD-L1) have been rapidly adopted in US clinical practice for first-line therapy of metastatic non-small cell lung cancer (NSCLC) since regulatory approval in October 2016, and a better understanding is needed of long-term outcomes of ICI therapy administered in real-world settings outside of clinical trials. Our aim was to describe long-term outcomes of first-line pembrolizumab monotherapy at US oncology practices for patients with metastatic NSCLC, PD-L1 expression ≥50%, and good performance status.

**Methods:**

This retrospective two-cohort study used technology-enabled abstraction of deidentified electronic health records (EHR cohort) plus enhanced manual chart review (spotlight cohort) to study adult patients with stage IV NSCLC, PD-L1 expression ≥50%, no documented *EGFR*/*ALK/ROS1* genomic aberration, and ECOG performance status 0–1 who initiated first-line pembrolizumab monotherapy from 1-November-2016 to 31-March-2020 (EHR cohort, with data cutoff 31-March-2021) or from 1-December-2016 to 30-November-2017 (spotlight cohort, with data cutoff 31-August-2020). Kaplan-Meier analysis was used to determine overall survival (OS; both cohorts) and, for the spotlight cohort, real-world progression-free survival (rwPFS) and real-world tumor response (rwTR).

**Results:**

The EHR cohort included 566 patients (298 [53%] men); the spotlight cohort included 228 (105 [46%] men); median age in both cohorts was 71. Median follow-up from pembrolizumab initiation to data cutoff was 35.1 months (range, 12.0–52.7) and 38.4 months (range, 33.1–44.9) in EHR and spotlight cohorts, respectively. Median OS was 19.6 months (95% CI, 16.6–24.3) and 21.1 months (95% CI, 16.2–28.9), respectively; 3-year OS rates were 36.2% and 38.2% in EHR and spotlight cohorts, respectively. In the spotlight cohort, median rwPFS was 7.3 months (95% CI, 5.7–9.2); 88 patients (38.6%; 95% CI, 32.2–45.2) experienced rwTR of complete or partial response. For 151/228 patients (66%) who discontinued pembrolizumab, the most common reasons were disease progression (70 [46%]) and therapy-related adverse effects (35 [23%]).

**Conclusions:**

Real-world outcomes remain consistent with outcomes observed in clinical trials, supporting long-term benefits of first-line pembrolizumab monotherapy for patients with metastatic NSCLC, PD-L1 expression ≥50%, and good performance status.

## 1 Introduction

Pembrolizumab was the first immune checkpoint inhibitor (ICI) of programmed cell death 1/programmed cell death ligand 1 (PD-1/PD-L1) approved in the United States (US) as a first-line treatment for metastatic non-small cell lung cancer (NSCLC). Supported by findings from the phase 3 randomized controlled trial, KEYNOTE-024 (1), this approval, in October 2016, was for pembrolizumab monotherapy in metastatic NSCLC with no *EGFR* or *ALK* genomic alterations and PD-L1 tumor proportion score (TPS) ≥50%, determined using a companion diagnostic test.

With 5 years of follow-up available for KEYNOTE-024, median overall survival (OS) was 26.3 months (95% CI, 18.3–40.4), and the 5-year survival rate was 32% in the pembrolizumab arm for enrolled patients with metastatic NSCLC (PD-L1 TPS ≥50%) and no sensitizing *EGFR* or *ALK* alterations (2). Moreover, with 4 years of follow-up in recent subgroup analyses of KEYNOTE-042, the median OS was 20.0 months (95% CI, 15.9–24.2), and the 3-year survival rate was 31%, in the PD-L1 TPS ≥50% subgroup of the pembrolizumab arm (3). In another trial, KEYNOTE-598, enrolling a similar patient population (metastatic NSCLC, PD-L1 TPS ≥50%, no *EGFR*/*ALK* alterations), patients who received first-line pembrolizumab monotherapy experienced median OS of 21.9 months (4).

Information from real-world settings is important to understand whether these long-term clinical trial findings apply to the wider population of patients who may not have been eligible or considered for trial participation (eg, because of comorbidities) and who are treated outside the controlled setting of these trials (5, 6). Cohort studies conducted before ICI approvals indicate that patients with metastatic NSCLC who received first-line therapy (most commonly platinum-based chemotherapy regimens) experienced a median OS of 10–11 months (7, 8). In one study, median OS was 9.7 months (95% CI, 9.1–10.3) for patients initiating first-line therapy in 2012–2015, thus before ICI approvals in the US, for stage IV NSCLC with no documented *EGFR* or *ALK* genomic alterations, not restricted to those with good performance status (8).

Since 2016, ICIs have been rapidly adopted in oncology practice as first-line therapies for advanced and metastatic NSCLC (9–11). In a recent retrospective database study of outcomes at US community oncology practices of 7746 patients initiating first-line therapy (March 2015–August 2018) for advanced NSCLC with no documented *EGFR*/*ALK* genomic alterations, 907 patients who initiated first-line ICI monotherapy experienced median OS of 19.9 months (95% CI, 16.6–24.1), superior to median OS with other recorded therapies (9). This cohort included 22% of patients with Eastern Cooperative Oncology Group (ECOG) performance status of 2 or greater (9).

In a prior study, we investigated outcomes for two cohorts of patients clinically similar to those in KEYNOTE-024 who received first-line pembrolizumab monotherapy in real-world US community oncology settings during the first 2 years after approval (2, 12). We observed that patients with good performance status who were prescribed first-line pembrolizumab monotherapy for metastatic NSCLC with PD-L1 expression ≥50% and no known *EGFR*, *ALK*, or *ROS1* alterations experienced clinical outcomes similar to those in the KEYNOTE trials, including OS, real-world progression-free survival (rwPFS), and real-world tumor response rate (rwTRR) for the patient cohort with median follow-up of 15.5 months (12). The objective of the present retrospective two-cohort study was to update those findings with longer follow-up using the same data source derived from electronic health records (EHRs) of patients at US oncology practices.

## 2 Methods

### 2.1 Data Source and Patients

The Flatiron Health database includes deidentified, longitudinal data from EHRs of patients with cancer at ~280 cancer clinics in the US, including approximately 800 sites of care (13). This nationally representative database is used frequently for cohort studies of advanced NSCLC and has previously been described in detail (8, 11, 14, 15). In brief, patient-level structured data, such as diagnosis and clinical visits, and unstructured data, such as physicians’ notes, are derived using technology-enabled data processing overseen by trained clinical abstractors. Our study also employed enhanced manual chart review to derive clinical outcomes data for the ‘spotlight cohort,’ described below.

Ethical approval of the study protocol was obtained from the Copernicus Group Institutional Review Board before study conduct, including a waiver of informed consent for working with deidentified data, and safeguards were in place to maintain data deidentification. Flatiron Health, Inc. did not participate in the analysis of the data.

Patients included in this study were drawn from the advanced NSCLC EHR database, which requires at least two clinical visits recorded on or after 1 January 2011, and pathologically confirmed evidence of advanced NSCLC on or after 1 January 2011. We selected two analytic cohorts of patients ≥18 years old with ECOG performance status of 0–1 who received first-line pembrolizumab monotherapy for stage IV NSCLC: (1.) The ‘EHR cohort’ included patients who initiated first-line pembrolizumab monotherapy from 1 November 2016 through 31 March 2020 (index period); data cutoff was on 31 March 2021, thus ensuring at least 1 year of potential follow-up time. (2.) The spotlight cohort included patients randomly selected from patients who initiated first-line pembrolizumab monotherapy from 1 December 2016 through 30 November 2017; data cutoff was 31 August 2020, thus ensuring almost 3 years of potential follow-up time. Other selection criteria for both cohorts were similar, including diagnosis of stage IV or recurrent metastatic NSCLC, tumor PD-L1 expression ≥50%, no known driver alterations (*EGFR*, *ALK*, or *ROS1*) and, for nonsquamous tumors, documented negative test results for both *EGFR* and *ALK* genomic alterations.

We excluded patients without structured database activity within 90 days of the metastatic diagnosis date (i.e., those possibly not receiving active clinical care), as well as patients with a record of clinical trial drug administered after the advanced NSCLC diagnosis. While some patients were likely included in both study cohorts, the rules protecting against patient reidentification prevented us from determining how many and who they were.

### 2.2 Study Variables

For each cohort, we summarized patient demographic characteristics, in addition to clinical characteristics available in the datasets, including smoking history, NSCLC histology, Charlson comorbidity index (CCI) score (derived from listed comorbidities (16)), and genomic testing results for *EGFR*, *ALK*, *ROS1*, *KRAS*, and *BRAF*. Lines of therapy were identified using Flatiron Health oncologist-defined business rules, with mapping of medication administrations and medication orders to lines of therapy (13).

Clinical outcomes determined for both cohorts included length of follow-up time and OS, the latter determined using death information according to the validated Flatiron Health composite mortality endpoint (17, 18). In addition, we summarized subsequent lines of therapy according to systemic anticancer regimen category.

For the spotlight cohort, manual chart abstraction was used to identify real-world progression (rwP) and real-world tumor response (rwTR) in order to determine rwPFS and rwTRR, respectively. The first episode in which the treating clinician concluded that there had been growth or worsening of NSCLC was identified as rwP, excluding events within 14 days of pembrolizumab initiation and including suspected pseudoprogression, which was defined as an increase in tumor size that the clinician recorded as possibly being an effect of ICI therapy (12, 19). Instead, rwTR was based on changes in NSCLC tumor burden indicated by radiology reports, excluding events within 30 days of pembrolizumab initiation, and mapped as complete response (CR), partial response (PR), stable disease, progressive disease (PD), and other (pseudoprogression, indeterminate, not documented) (12, 20). Both of these endpoints have been recently described and characterized using Flatiron Health advanced NSCLC datasets (12, 19, 20). For the present study, the rwTRR determination was defined as the proportion of patients with at least one CR or PR assessment followed by a subsequent assessment of CR, PR, or stable disease during first-line pembrolizumab monotherapy.

Manual chart abstraction was also used to determine the reasons for pembrolizumab discontinuation when available. Pembrolizumab was considered discontinued when clinical notes explicitly stated discontinuation or with a gap >60 days in pembrolizumab administration. Although adverse events were not actively solicited in this study, we collected any reports of individual adverse events identified during manual chart review, and all were reported as related to pembrolizumab.

### 2.3 Statistical Analyses

We used summary statistics to describe baseline demographic and clinical characteristics and subsequent lines of therapy for each cohort, in addition to rwTR categories for patients in the spotlight cohort.

The Kaplan-Meier method was used to estimate time-to-event analyses, including OS and rwPFS, beginning from pembrolizumab initiation. We determined OS as (date of death - start date of pembrolizumab) + 1 day, setting the date of death to the 15^th^ of the month because only month and year of death were provided to maintain patient anonymity. Patients with no recorded date of death were censored at the end of the dataset or at their last recorded activity in the dataset, whichever occurred first. Similarly, rwPFS was determined from the start of pembrolizumab to first recorded rwP; patients with no rwP were censored at their last recorded activity or at the initiation of a new line of therapy. In a sensitivity analysis, we excluded suspected pseudoprogression as an event.

The Kaplan-Meier method was also used to estimate median real-world time on treatment (rwToT) for second- and third-line ICI therapy, as previously described (21). In brief, rwToT was calculated as the (date of the last ICI dose - date of first ICI dose) + 1 day and defined as the length of time between first and last administration dates before discontinuation. Patients who had a record of initiating the next line of therapy, or who died while receiving the ICI therapy, were considered discontinued at their last administration date. If none of these events were identified, then having a gap of ≥120 days between the last administration date and last known activity date in the dataset was considered discontinued at the last administration date. If none of the discontinuation criteria were met, patients were considered censored at their last administration date.

A formal calculation of sample size and power was not performed because of the descriptive nature of the study. Statistical analyses were performed using SAS software, version 9.4 (SAS Institute, Cary, NC).

## 3 Results

### 3.1 Patients

**Figure 1:**
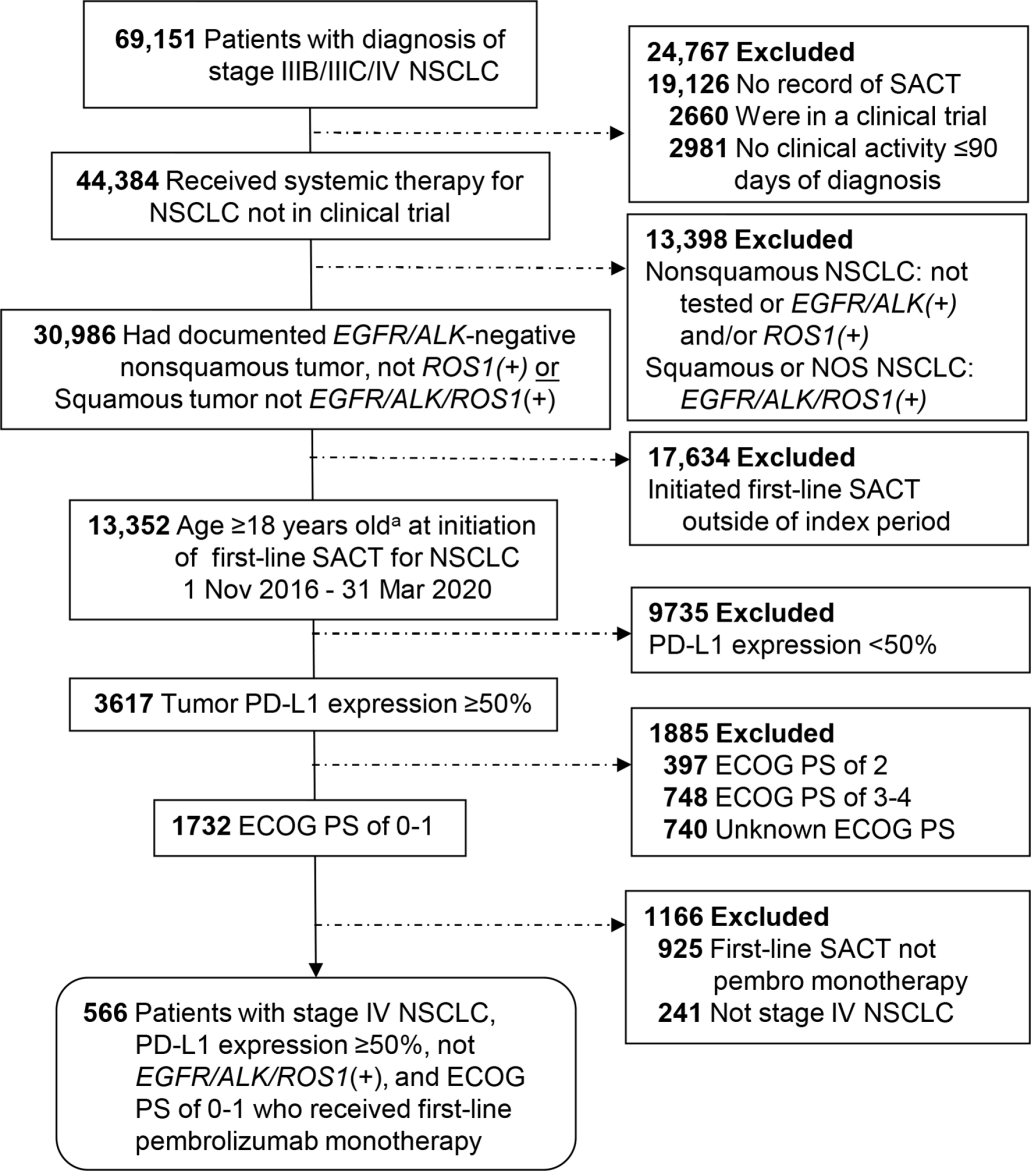
Patient selection for the EHR cohort from the Flatiron Health Database. ^a^No patients were excluded for being <18 years of age. ECOG PS, Eastern Cooperative Oncology Group performance status; NSCLC, non-small cell lung cancer; PD-L1, programmed cell death-ligand 1; SACT, systemic anticancer therapy.

**Figure 2:**
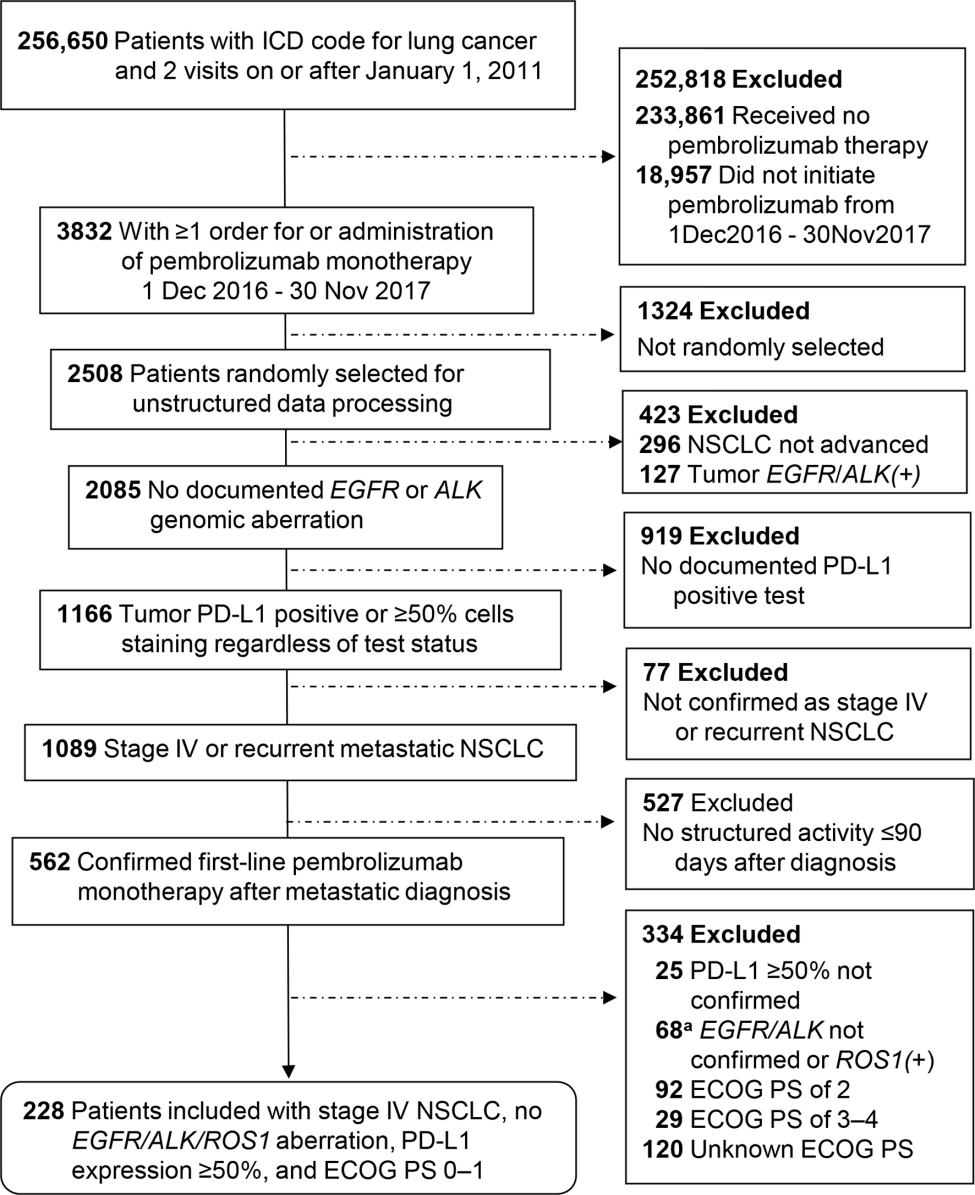
Patient selection for the spotlight cohort from the Flatiron Health Database. ^a^Exclusion for lacking confirmation of negative test status for *EGFR/ALK* genomic aberration applied only to nonsquamous tumors. ECOG PS, Eastern Cooperative Oncology Group performance status; PD-L1, programmed cell death-ligand 1.

A total of 566 patients who initiated first-line pembrolizumab monotherapy from November 2016 through March 2020 were included in the EHR cohort, and 228 patients initiating first-line pembrolizumab monotherapy from December 2016 through November 2017 were included in the spotlight cohort. The details of patient selection are depicted in **Figures 1** and **2**.

In the EHR cohort slightly over half of patients were men (53%), while in the spotlight cohort 46% were men (**Table 1**). The median age in both cohorts was 71 years, with approximately one-third of patients aged 75 years or older. Most patients in each cohort were White (76–79%), and >90% were current or former smokers. The two cohorts included similar proportions of patients by NSCLC histology, including approximately two-thirds with nonsquamous and one-quarter with squamous histology (**Table 1**). Of the nonsquamous tumors, 26% and 24% in EHR and spotlight cohorts were *KRAS* positive, respectively, and 7% and 6%, respectively, were *BRAF* mutant (**Table 1**).

**Table 1:** Baseline demographic and clinical characteristics of patients with stage IV NSCLC, PD-L1 expression ≥50%.

Variable	EHR cohort (n = 566)	Spotlight cohort (n = 228)
Male sex	298 (52.7)	105 (46.1)
Age, median (range), y	71 (38-84)	71 (46-82)
<75 years	359 (63.4)	138 (60.5)
≥75 years	207 (36.6)	90 (39.5)
Race data available^a^	505 (89.2)	209 (91.7)
White	383 (75.8)	165 (78.9)
Black	49 (9.7)	18 (8.6)
Asian	17 (3.4)	6 (2.9)
Other race	56 (11.1)	20 (9.6)
Current/former smoker	524 (92.6)	209 (91.7)
No smoking history	42 (7.4)	19 (8.3)
CCI score, mean (SD)	5.1 (3.1)	3.1 (3.2)
Median (range)	4 (0-13)	2 (0-10)
NSCLC histology		
Nonsquamous	405 (71.6)	156 (68.4)
NSCLC histology NOS	28 (4.9)	12 (5.3)
Squamous	133 (23.5)	60 (26.3)
ECOG performance status		
0	201 (35.5)	95 (41.7)
1	365 (64.5)	133 (58.3)
Record of brain metastases^b^	69 (12.2)	17 (7.5)
US CB region, data available^a^	552 (97.5)	219 (96.1)
Midwest	127 (23.0)	51 (23.3)
Northeast	105 (19.0)	58 (26.5)
South	249 (45.1)	84 (38.4)
West	71 (12.9)	26 (11.9)
Community oncology clinic	558 (98.6)	223 (97.8)
Academic oncology clinic	8 (1.4)	5 (2.2)
Index year		
2016	14 (2.5)	7 (3.1)
2017	210 (37.1)	221 (96.9)
2018	159 (28.1)	0
2019	142 (25.1)	0
2020	41 (7.2)	0
*BRAF* mutation status (nonsquamous only), N	*405*	*156*
Positive^c^	27 (6.7)	9 (5.8)
Wild type	249 (61.5)	68 (43.6)
Indeterminate, unknown, pending, untested	129 (31.9)	79 (50.6)
*KRAS* mutation status (nonsquamous only), N	*405*	*156*
Positive^c^	107 (26.4)	37 (23.7)
Wild type	125 (30.9)	38 (24.4)
Indeterminate, unknown, pending, untested	173 (42.7)	81 (51.9)
IHC clone, data available^d^	514 (90.8)	201 (88.2)
22C3c	474 (92.2)	187 (93.0)
SP263	17 (3.3)	9 (4.5)
Other	23 (4.5)	5 (2.5)

Data are presented as n (%) of patients unless otherwise indicated. Percentages may not add up to 100% because of rounding.

a Percentages for race and US CB region represent the percentages of patients with available data.

b Information about prior treatment of brain metastases was not available.

c Positive biomarker results at any time (“ever positive”) were included.

d Of the 22C3 IHC assays, 455/474 (96.0%) and 182/187 (97.3%) in EHR and Spotlight cohorts, respectively, used the PD-L1 IHC 22C3 pharmDx (Agilent Technologies, Carpinteria, CA; pembrolizumab companion diagnostic).

CCI, Charlson comorbidity index; ECOG, Eastern Cooperative Oncology Group; EHR, electronic health record; IHC, immunohistochemistry; index year, year of pembrolizumab initiation; NSCLC histology NOS, non-small cell lung cancer histology not otherwise specified; US CB, United States Census Bureau.

The geographical distribution of oncology clinics was similar in the two cohorts, and all but 8 and 5 patients in EHR and spotlight cohorts, respectively, were seen at community (rather than academic) oncology clinics (**Table 1**).

### 3.2 Clinical Outcomes: EHR and Spotlight Cohorts

Median observed follow-up ending at data cutoff in EHR and spotlight cohorts was 35.1 and 38.4 months respectively, while median patient follow-up was 16.5 and 25.7 months, respectively (**Table 2**). Patients in the EHR cohort received a median of 9 cycles of pembrolizumab (range, 1–73), while those in the spotlight cohort received a median of 11 cycles of pembrolizumab (range, 1–61).

**Table 2:** Outcomes with first-line pembrolizumab monotherapy in the real-world oncology and clinical trial settings.

Variable	Real-world cohorts		Clinical trials	
	EHR cohort^a^ (n = 566)	Spotlight cohort^a^ (n = 228)	KEYNOTE-024 (2)^b^ (n = 154)	KEYNOTE-042 (3)^b^ (n = 299)
Observed follow-up, median (range), mo^c^	35.1 (12.0-52.7)	38.4 (33.1-44.9)	59.9 (55.1-68.4)	46.9 (35.8‒62.1)
Patient follow-up, median (range), mo^c^	16.5 (<0.1-52.6)	25.7 (1 day-44.3)	–	–
Real-world TRR (rwTRR)/ORR, n^d^	–	88	71	–
% (95% CI)	–	38.6 (32.2-45.2)	46.1 (38.1-54.3)	–
Time to response, median (range), mo	–	3.2 (1.5-34.4)	2.1 (1.4-14.6)	–
Duration of response, median (range), mo		22.2 (1.4+ to 37.2+)^e^	29.1 (2.2-60.8+)	
Real-world PFS (rwPFS)/PFS				
Events, n (%)	–	184 (80.7)	126 (81.8)	–
rwPFS, median (95% CI), mo	–	7.3 (5.7-9.2)	7.7 (6.1-10.2)	6.5 (5.9-8.6)
12-month rwPFS, % (95% CI)	–	39.3 (32.8-45.7)	–	–
24-month rwPFS, % (95% CI)	–	25.9 (20.2-31.9)	–	–
36-month rwPFS, % (95% CI)	–	14.3 (9.7-19.7)	22.8 (16.3-29.9)	14.5 (10.5-19.0)
Overall survival (OS), N	566	228	154	299
Events, n (%)	322 (56.9)	134 (58.8)	103 (66.9)	219 (73)
OS, median (95% CI), mo	19.6 (16.6-24.3)	21.1 (16.2-28.9)	26.3 (18.3-40.4)	20.0 (15.9-24.2)
12-month survival, % (95% CI)	59.8 (55.5-63.7)	64.2 (57.5-70.2)	–	–
24-month survival, % (95% CI)	45.7 (41.2-50.0)	49.4 (42.5-55.8)	–	–
36-month survival, % (95% CI)	36.2 (31.5-40.9)	38.2 (31.4-45)	43.7	31.3 (26.1-36.6)

a EHR cohort data cutoff, 31 March 2021; spotlight cohort data cutoff, 31 August 2020.

b Investigator-assessed tumor response and PFS in KEYNOTE-024 are reported. Results from KEYNOTE-042 are reported for patients with PD-L1 TPS ≥50% with locally advanced or metastatic NSCLC, the majority of whom had metastatic NSCLC (n=275; 92%).

c Observed (theoretical) follow-up was defined as the duration of follow-up from pembrolizumab initiation to database cutoff. Patient follow-up was defined as time from pembrolizumab initiation to the date of death or data cutoff, whichever occurred first.

d rwTRR refers to the real-world tumor response rate, defined as the proportion of patients with at least one complete response (CR) or partial response (PR) assessment followed by a subsequent assessment of CR, PR, or stable disease during first-line pembrolizumab monotherapy. Analysis of time to response is based on patients with a best rwTR of CR or PR.

e + indicates ongoing response.

EHR, electronic health record; mo, months; NR, not reached; ORR, objective response rate; PFS, progression-free survival; TRR, tumor response rate.

**Figure 3:**
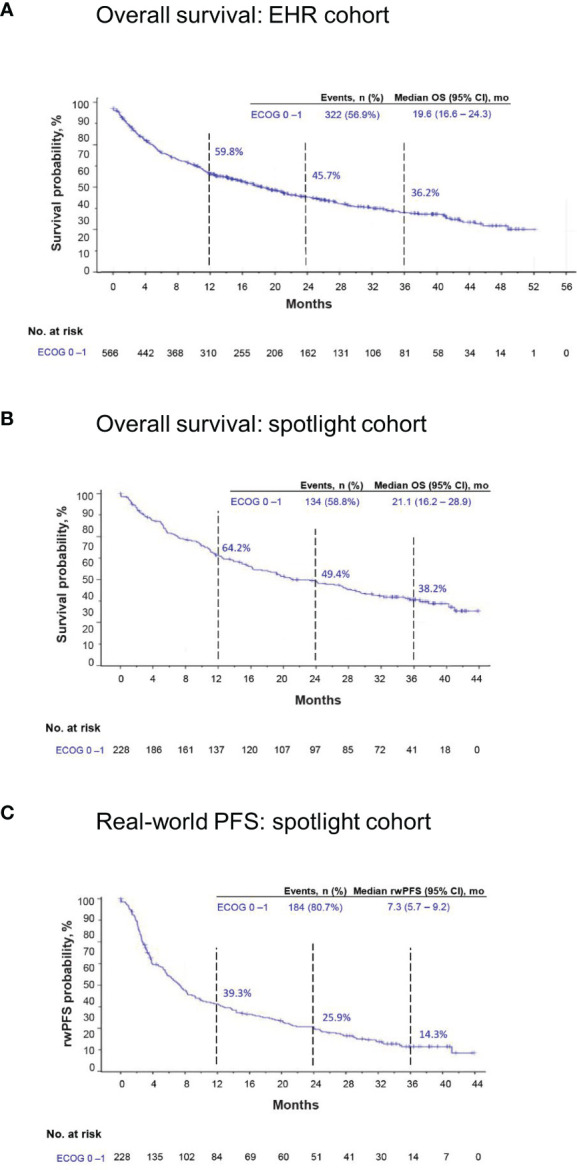
Kaplan-Meier estimates of overall survival (OS) in the EHR and spotlight cohorts and real-world progression-free survival (rwPFS) in the spotlight cohort.EHR, electronic health record; mo, months. **(A)** OS in the EHR cohort. **(B)** OS in the spotlight cohort. **(C)** rwPFS in the spotlight cohort.

At data cutoff, 322 patients (57%) had died in the EHR cohort, and 134 patients (59%) had died in the spotlight cohort. Median OS was 19.6 months (95% CI, 16.6–24.3) and 21.1 months (95% CI, 16.2–28.9) in EHR and spotlight cohorts, respectively; Kaplan-Meier estimates of OS at 3 years were 36.2% and 38.2%, respectively (**Table 2**; **Figure 3**).

Second-line systemic therapy, most commonly platinum-based chemotherapy or immunotherapy (ICI monotherapy or ICI-chemotherapy combination), was administered to 182 patients (32%) in the EHR cohort, of whom 63 (35%) received third-line and 16/63 (25%) received fourth-line therapy (details in **Supplementary Table 1**). Immunotherapy was administered in second line to 72/182 patients (40%), including 16 patients (22%) who received pembrolizumab monotherapy, to 19 patients (30%) in third line (4 pembrolizumab monotherapy), to 3 patients (19%) in fourth line, and to 3 (50%) in fifth line.

In the spotlight cohort, 87 patients (38%) received second-line therapy, of whom 26 (30%) also received third-line therapy; only 7 and 3 patients received fourth- and fifth-line therapy, respectively (**Supplementary Table 2**). The pattern of second-line therapies was similar to that for the EHR cohort, with the most common being platinum-based chemotherapy and ICIs, the latter administered in second line to 28/87 patients (32%), including 8/28 patients (29%) who received pembrolizumab monotherapy. Eight patients (31%) in third line (1 pembrolizumab monotherapy), and 4 patients (57%) in fourth line, were treated with ICIs. The median rwToT for second-line ICI therapy was 5.8 months (95% CI, 1.4–12.9) and for third-line ICI therapy was 5.6 months (95% CI, 0 to not assessable).

#### 3.2.1 Spotlight Cohort: Outcomes Captured Using Enhanced Manual Chart Review

Additional outcomes for the spotlight cohort are summarized in **Table 2**. Median rwPFS was 7.3 months (95% CI, 5.7–9.2 months), and 184 patients (81%) experienced an episode of rwP or death from any cause (**Figure 3C**). In a sensitivity analysis excluding suspected pseudoprogression, 182 patients (80%) experienced rwP, and median rwPFS was 8.6 months (95% CI, 6.8–11.2).

A total of 17 patients (7%) had a best rwTR assessed as CR, and 71 patients (31%) were assessed as having PR (**Table 3**), for a rwTRR of 38.6% (95% CI, 32.2–45.2), and a total of 122 patients (54%) with disease control (CR + PR + stable disease).

**Table 3:** Best real-world tumor response (rwTR) to first-line pembrolizumab monotherapy: Spotlight cohort.

	Spotlight^a^ (n = 228)	KEYNOTE-024^b^ (n = 154)
Complete response (CR)	17 (7.5)	7 (4.5)
Partial response (PR)	71 (31.1)	64 (41.6)
Stable disease	34 (14.9)	37 (24.0)
Progressive disease (PD)	50 (21.9)	35 (22.7)
No evaluable assessment	56 (24.6)	0
Indeterminate	1 (0.4)	–
Pseudoprogression^c^	7 (3.1)	–
Not documented/no assessment	51 (22.4)	11 (7.1)

Data are presented as n (%) of patients.

a rwTR determination was based on changes in NSCLC tumor burden indicated by radiology reports. For patients with multiple rwTR assessments, the best response was used to classify the patient (CR>PR>stable disease>PD). Patients without an evaluable assessment (no CR, PR, stable disease, or PD) could be counted >1 time in the subcategories of “no evaluable assessment”.

b KEYNOTE-024 results determined using RECIST 1.1 criteria by investigatory review (2).

c Pseudoprogression was defined as an increase in tumor size that the clinician recorded as possibly being an effect of ICI therapy.

Two-thirds of patients (151; 66%) in the spotlight cohort had discontinued pembrolizumab at data cutoff (**Table 4**). The most common reason for discontinuation was disease progression (70/151; 46%), followed by adverse effect of therapy (35/151; 23%). Of the 33 patients who subsequently received second- and/or third-line ICI therapy, 8 discontinued first-line pembrolizumab because of disease progression, 6 because of adverse effects, and 5 because of disease-related symptoms (**Supplementary Table 3**).

**Table 4:** Summary of reasons for pembrolizumab discontinuation: Spotlight cohort.

	Spotlight (n = 228)
Discontinued, n (%)	151 (66.2)
Reasons for discontinuation, n (%)	*N=151^a^*
Progression	70 (46.4)
Adverse effect of therapy	35 (23.2)
Disease-related symptoms not due to therapy	23 (15.2)
Patient request	6 (4)
Completed treatment	5 (3.3)
No evidence of disease	2 (1.3)
Other^b^	13 (8.6)
Unknown	1 (0.7)

a Patients could have more than one reason for discontinuation.

b For patients with ongoing treatment until the time of death, the reason recorded was “Other” to comply with data deidentification requirements.

No serious adverse events were identified during manual chart review (**Supplementary Table 4**).

## 4 Discussion

This study enlarges and extends the observational period for two real-world patient cohorts treated with pembrolizumab monotherapy identified as being clinically similar to patients enrolled in KEYNOTE-024. The minimum potential follow-up after pembrolizumab initiation to data cutoff was 6 months in our prior study (12), and, in the present study, was extended to 1 year for the EHR cohort and almost 3 years for the spotlight cohort. Median OS was 19.6 and 21.1 months in EHR and spotlight cohorts, respectively, while median OS with pembrolizumab monotherapy in KEYNOTE-024, -042, and -598 was 26.3, 20.0, and 21.9 months, respectively (2–4), We observed 3-year Kaplan-Meier estimated OS rates of 36% and 38% in EHR and spotlight cohorts, respectively, whereas 3-year OS rates in KEYNOTE-024 and KEYNOTE-042 (PD-L1 TPS ≥50% population) were 44% and 31%, respectively (2, 3).

There was no comparator included in the present study; however, the median OS for both EHR and spotlight cohorts was substantially higher than the median OS of patients who received chemotherapy in KEYNOTE-024 (13.4 months) (2). Tumor characteristics were similar, but our real-world populations included proportionately more women and older patients relative to the patient populations in KEYNOTE trials (2, 22), as we reported previously (12). There were likely other differences as well that were not captured *via* retrospective EHR review: for example, we could not reliably capture pretreatment of brain metastases or determine whether patients had a minimum life expectancy of 3 months, as required in KEYNOTE-024 and -042 (1, 22). Subsequent therapy use in both EHR and spotlight cohorts (32% and 38%) was lower than in the KEYNOTE-024 5-year data (53%). However, we observed that in these two real-world cohorts, ICIs, as monotherapy or in combination with chemotherapy, were used as a second- or third-line therapy, and administered for a median rwToT of almost 6 months, among 30–40% of patients receiving a subsequent treatment.

Several other observational studies from the US, Europe, and Israel have been published recently that evaluated outcomes with first-line pembrolizumab or other ICI monotherapy for similar patient populations with advanced NSCLC, PD-L1 expression ≥50%, and no genomic alterations (23–27). There was some variability in patient populations and length of follow-up time, and survival in these studies for patients with good performance status (ECOG 0–1) also varied, with reported median OS of 18.6 months (26), 22.1 months (28), 22.8 months (23), 28.7 months (25), and not reached (27). In a large European multicenter study of first-line pembrolizumab monotherapy (n=1026), the objective response rate determined by investigators using RECIST (v1.1) criteria (29) was 48.1% for 756 evaluable patients with ECOG 0–1 (23), greater than the 39% rwTRR determined in our study, although the means of rwTR determination *via* manual chart review is not directly comparable with the application of RECIST criteria.

Our study had the advantages of a large patient population and, for the spotlight cohort, long follow-up time. However, while the clinical similarity of our patient population to KEYNOTE trial populations enabled us to examine outcomes relative to those in clinical trials, our ability to investigate potential prognostic factors was limited. Results of a recent study also drawing on the Flatiron Health database indicated that among patients with nonsquamous NSCLC and PD-L1 expression ≥50% treated with first-line pembrolizumab monotherapy, never-smokers had shorter rwPFS and OS than smokers (28), similar to findings in clinical trials and other observational studies (30, 31). Prior cohort studies have identified several independent predictors of shorter OS, including ECOG performance status of ≥2, bone metastases, liver metastases, and baseline steroids (23, 25). Conversely, tumor PD-L1 expression ≥90% was associated with longer OS in multivariable analyses of a large cohort of patients with metastatic NSCLC and PD-L1 expression ≥50% treated with first-line pembrolizumab monotherapy (23). We note that a recent study evaluating outcomes of first-line pembrolizumab monotherapy for a less selective, older US population (≥66 years) with advanced NSCLC reported a median OS of only 11.4 months (32), 15 months less than that in KEYNOTE-024 (2) and considerably shorter than the median OS determined for each of our study cohorts. As the authors of that study observed, the administrative claims data they used lack many important prognostic and predictive baseline factors, including performance status, targetable mutations, and PD-L1 status, to allow stratified analyses by these baseline factors to further explain the results (32). Clearly, further study is needed of potential prognostic factors, including biomarkers, for more heterogeneous populations with metastatic NSCLC treated with pembrolizumab as monotherapy, or in combination with chemotherapy, to inform clinical treatment decisions.

Other strengths of the present study include the use of a well-regarded, well-curated EHR database. Manual chart review for the randomly selected spotlight cohort enabled the determination of rwPFS, rwTR, and reasons for pembrolizumab discontinuation. The curation of rwPFS and rwTRR endpoints from EHR datasets has recently been described and characterized as reliable and clinically relevant (19, 20).

Continued follow-up of these real-world cohorts will be of interest. In addition, as we observed that a non-negligible proportion of patients were treated with an ICI following first-line pembrolizumab, it will be of interest to understand the long-term real-world outcomes of these patients rechallenged with an ICI in a later line of therapy.

### 4.1 Limitations

The limitations of retrospective data evaluation are applicable to this study, including the potential for missing or inaccurately recorded data. Attrition in cohort selection was primarily due to missing eligibility-related data, for example, missing ECOG performance status, genomic testing results for *EGFR/ALK* alterations, or PD-L1 testing results. In addition, even with manual chart review for the spotlight cohort, some outcome data, such as information to determine rwTR and reasons for pembrolizumab discontinuation, were missing for some patients.

### 4.2 Conclusions

With long-term follow-up, real-world outcomes with first-line pembrolizumab monotherapy remain consistent with outcomes observed in phase 3 pivotal clinical trials for patients with metastatic NSCLC, PD-L1 expression ≥50%, no known tumor genomic alterations, and good performance status. These findings support the long-term benefits of first-line pembrolizumab monotherapy for this patient population.

## Data Availability Statement

The data that support the findings of this study have been originated by Flatiron Health, Inc. These deidentified data may be made available upon request and are subject to a license agreement with Flatiron Health; interested researchers should contact DataAccess@flatiron.com to determine licensing terms. Requests to access the datasets should be directed to Flatiron Health DataAccess@flatiron.com.

## Ethics Statement

The studies involving human participants were reviewed and approved by Copernicus Group Institutional Review Board. Written informed consent for participation was not required for this study in accordance with the national legislation and the institutional requirements.

## Author Contributions

VV, XH, LY, and TB developed the study concept and design. LY conducted the statistical analysis. VV, XH, LY, MCP, and TB interpreted the data and critically revised the manuscript for important intellectual content. All authors contributed to the article and approved the submitted version.

## Conflict of Interest

All authors, including those employed by Merck Sharp & Dohme Corp., a subsidiary of Merck & Co., Inc., Kenilworth, NJ, USA, participated in the data interpretation, writing of the report, and the decision to submit the article for publication. VV reports serving in an advisory and/or consultant role for Merck, Bristol-Myers Squibb, AstraZeneca, Novartis, Amgen, Bayer, and Foundation Medicine. XH, MCP, and TB are full-time employees of Merck Sharp & Dohme, Corp., a subsidiary of Merck & Co., Inc., Kenilworth, NJ, USA and own stock of Merck & Co., Inc., Kenilworth, NJ, USA. LY was a full-time employee of Merck Sharp & Dohme, Corp., a subsidiary of Merck & Co., Inc., Kenilworth, NJ, USA at the time of the study.

This study received funding from Merck Sharp & Dohme Corp., a subsidiary of Merck & Co., Inc., Kenilworth, NJ, USA. The funder had the following involvement with the study: the funder played a role in the design and conduct of the study; collection, management, analysis, and interpretation of the data; preparation, review, and approval of the manuscript; and the decision to submit the manuscript for publication.

## Publisher’s Note

All claims expressed in this article are solely those of the authors and do not necessarily represent those of their affiliated organizations, or those of the publisher, the editors and the reviewers. Any product that may be evaluated in this article, or claim that may be made by its manufacturer, is not guaranteed or endorsed by the publisher.
