# Supplementary material for: Long-Term Real-World Outcomes of First-Line Pembrolizumab Monotherapy for Metastatic Non-Small Cell Lung Cancer With ≥50% Expression of Programmed Cell Death-Ligand 1
Source: Front Oncol. 2022 Mar 25;12:834761. doi: 10.3389/fonc.2022.834761 (PMC8990758; doi:10.3389/fonc.2022.834761)
Supplement: Supplementary file 1 [file DataSheet_1.zip › Supplementary Material/Tables 1 - 4.DOCX]

Supplementary Material

**Supplementary Table 1 |** Subsequent lines of systemic anticancer therapy in the EHR cohort: lines 2-7.

| **Regimen by Treatment Line** | **EHR Cohort**  **(N = 566)** |
| --- | --- |
| **Systemic Therapy Line 2** | **182 (32.2)** |
| **Line 2: Anti-PD-1/PD-L1-based therapy** | **72 (39.6)** |
| Carboplatin, pembrolizumab, pemetrexed | 24 (33.3) |
| Pembrolizumab | 16 (22.2) |
| Carboplatin, paclitaxel, pembrolizumab | 10 (13.9) |
| Atezolizumab | 3 (4.2) |
| Nivolumab | 3 (4.2) |
| Pembrolizumab, pemetrexed | 2 (2.8) |
| Anastrozole, carboplatin, pembrolizumab, pemetrexed | 1 (1.4) |
| Anastrozole, pembrolizumab | 1 (1.4) |
| Atezolizumab, carboplatin, paclitaxel | 1 (1.4) |
| Bevacizumab, pembrolizumab | 1 (1.4) |
| Bevacizumab, pembrolizumab, pemetrexed | 1 (1.4) |
| Capecitabine, pembrolizumab | 1 (1.4) |
| Carboplatin, gemcitabine, pembrolizumab, pemetrexed | 1 (1.4) |
| Carboplatin, ipilimumab, nivolumab, pemetrexed | 1 (1.4) |
| Carboplatin, paclitaxel protein-bound, pembrolizumab | 1 (1.4) |
| Exemestane, pembrolizumab | 1 (1.4) |
| Hydroxyurea, pembrolizumab | 1 (1.4) |
| Imatinib, pembrolizumab | 1 (1.4) |
| Ipilimumab, nivolumab, pembrolizumab | 1 (1.4) |
| Medroxyprogesterone, pembrolizumab | 1 (1.4) |
| **Line 2: Anti-VEGF-based therapy** | **18 (9.9)** |
| Bevacizumab, carboplatin, pemetrexed | 13 (72.2) |
| Bevacizumab-awwb, carboplatin, pemetrexed | 2 (11.1) |
| Bevacizumab, cisplatin, pemetrexed | 1 (5.6) |
| Bevacizumab, gemcitabine | 1 (5.6) |
| Bevacizumab-bvzr, carboplatin, paclitaxel | 1 (5.6) |
| **Line 2: Platinum-based chemotherapy combination^a^** | **66 (36.3)** |
| Carboplatin, pemetrexed | 36 (54.5) |
| Carboplatin, paclitaxel | 12 (18.2) |
| Carboplatin, paclitaxel protein-bound | 10 (15.2) |
| Carboplatin, gemcitabine | 6 (9.1) |
| Carboplatin, etoposide | 1 (1.5) |
| Cisplatin, gemcitabine | 1 (1.5) |
| **Line 2: Non-platinum-based chemo combination** | **1 (0.5)** |
| Gemcitabine, paclitaxel | 1 (100) |
| **Line 2: Single agent chemotherapy** | **17 (9.3)** |
| Pemetrexed | 10 (58.8) |
| Docetaxel | 4 (23.5) |
| Gemcitabine | 1 (5.9) |
| Paclitaxel | 1 (5.9) |
| Vinorelbine | 1 (5.9) |
| **Line 2: Other therapy** | **8 (4.4)** |
| Dabrafenib, trametinib | 3 (37.5) |
| Ado-trastuzumab emtansine | 1 (12.5) |
| Alectinib | 1 (12.5) |
| Capmatinib | 1 (12.5) |
| Leuprolide | 1 (12.5) |
| Methotrexate | 1 (12.5) |
| **Systemic Therapy Line 3** | **63 (11.1)** |
| **Line 3: Anti-PD-1/PD-L1-based therapy** | **19 (30.2)** |
| Nivolumab | 4 (21.1) |
| Pembrolizumab | 4 (21.1) |
| Carboplatin, pembrolizumab, pemetrexed | 3 (15.8) |
| Atezolizumab | 1 (5.3) |
| Atezolizumab, bevacizumab-awwb, carboplatin, paclitaxel | 1 (5.3) |
| Bevacizumab-awwb, carboplatin, pembrolizumab, pemetrexed | 1 (5.3) |
| Capecitabine, pembrolizumab | 1 (5.3) |
| Carboplatin, paclitaxel, pembrolizumab | 1 (5.3) |
| Fluorouracil, irinotecan, pembrolizumab | 1 (5.3) |
| Nivolumab, temozolomide | 1 (5.3) |
| Pembrolizumab, pemetrexed | 1 (5.3) |
| **Line 3: Anti-VEGF-based therapy** | **12 (19.0)** |
| Docetaxel, ramucirumab | 6 (50.0) |
| Bevacizumab | 1 (8.3) |
| Bevacizumab, carboplatin, gemcitabine | 1 (8.3) |
| Bevacizumab-awwb, carboplatin, pemetrexed | 1 (8.3) |
| Bevacizumab-awwb, pemetrexed | 1 (8.3) |
| Bevacizumab-bvzr, carboplatin, pemetrexed | 1 (8.3) |
| Paclitaxel, ramucirumab | 1 (8.3) |
| **Line 3: Platinum-based chemotherapy combination^a^** | **9 (14.3)** |
| Carboplatin, paclitaxel | 6 (66.7) |
| Carboplatin, pemetrexed | 2 (22.2) |
| Carboplatin, hydroxyurea, pemetrexed | 1 (11.1) |
| **Line 3: Non-platinum-based chemo combination** | **1 (1.6)** |
| Docetaxel, paclitaxel protein-bound | 1 (100) |
| **Line 3: Single agent chemotherapy** | **19 (30.2)** |
| Gemcitabine | 7 (36.8) |
| Docetaxel | 5 (26.3) |
| Pemetrexed | 4 (21.1) |
| Vinorelbine | 3 (15.8) |
| **Line 3: Other therapy** | **3 (4.8)** |
| Afatinib | 1 (33.3) |
| Alectinib, carboplatin, paclitaxel | 1 (33.3) |
| Brigatinib | 1 (33.3) |
| **Systemic Therapy Line 4** | **16 (2.8)** |
| **Line 4: Anti-PD-1/PD-L1-based therapy** | **3 (18.8)** |
| Atezolizumab | 1 (33.3) |
| Docetaxel, irinotecan, pembrolizumab | 1 (33.3) |
| Ipilimumab, nivolumab | 1 (33.3) |
| **Line 4: Anti-VEGF-based therapy** | **3 (18.8)** |
| Bevacizumab-awwb, carboplatin, paclitaxel protein-bound | 1 (33.3) |
| Bevacizumab-bvzr | 1 (33.3) |
| Docetaxel, ramucirumab | 1 (33.3) |
| **Line 4: Platinum-based chemotherapy combination^a^** | **3 (18.8)** |
| Carboplatin, etoposide | 1 (33.3) |
| Carboplatin, gemcitabine | 1 (33.3) |
| Carboplatin, gemcitabine, temozolomide | 1 (33.3) |
| **Line 4: Single agent chemotherapy** | **6 (37.5)** |
| Gemcitabine | 3 (50.0) |
| Docetaxel | 1 (16.7) |
| Pemetrexed | 1 (16.7) |
| Vinorelbine | 1 (16.7) |
| **Line 4: Other therapy** | **1 (6.3)** |
| Regorafenib | 1 (100) |
| **Systemic Therapy Line 5** | **6 (1.1)** |
| **Line 5: Anti-PD-1/PD-L1-based therapy** | **3 (50.0)** |
| Atezolizumab | 1 (33.3) |
| Capecitabine, epirubicin, pembrolizumab | 1 (33.3) |
| Nivolumab | 1 (33.3) |
| **Line 5: Anti-VEGF-based therapy** | **1 (16.7)** |
| Ramucirumab | 1 (100) |
| **Line 5: Platinum-based chemotherapy combination^a^** | **1 (16.7)** |
| Carboplatin, paclitaxel | 1 (100) |
| **Line 5: Single agent chemotherapy** | **1 (16.7)** |
| Gemcitabine | 1 (100) |
| **Systemic Therapy Line 6** | **3 (0.5)** |
| **Line 6: Anti-PD-1/PD-L1-based therapy** | **1 (33.3)** |
| Cisplatin, irinotecan, pembrolizumab | 1 (100) |
| **Line 6: Anti-VEGF-based therapy** | **1 (33.3)** |
| Bevacizumab-bvzr, paclitaxel protein-bound | 1 (100) |
| **Line 6: Other therapy** | **1 (33.3)** |
| Dabrafenib, trametinib | 1 (100) |
| **Systemic Therapy Line 7** | **1 (0.2)** |
| **Line 7: Other therapy** | **1 (100)** |
| Dabrafenib, hydroxyurea, trametinib | 1 (100) |

Data are n (%).

^a^ Platinum-based chemotherapy combinations without anti-VEGF agent.

PD1/PD-L1, programmed death 1/programmed death-ligand 1; VEGF, vascular endothelial growth factor.

**Supplementary Table 2 |** Subsequent lines of systemic anticancer therapy in the spotlight cohort: lines 2-5.

| **Regimen by Treatment Line** | **Spotlight Cohort (N = 228)** |
| --- | --- |
| **Systemic Therapy Line 2** | **87 (38.2)** |
| **Line 2: Anti-PD-1/PD-L1-based therapy** | **28 (32.2)** |
| Pembrolizumab | 8 (28.6) |
| Carboplatin, pembrolizumab, pemetrexed | 9 (32.1) |
| Carboplatin, paclitaxel, pembrolizumab | 3 (10.7) |
| Nivolumab | 1 (3.6) |
| Bevacizumab, pembrolizumab | 2 (7.1) |
| Gemcitabine, pembrolizumab | 1 (3.6) |
| Atezolizumab, carboplatin, paclitaxel | 1 (3.6) |
| Bevacizumab, pembrolizumab, pemetrexed | 1 (3.6) |
| Pembrolizumab, vinorelbine | 1 (3.6) |
| Docetaxel, pembrolizumab | 1 (3.6) |
| **Line 2: Anti-VEGF-based therapy** | **12 (13.8)** |
| Bevacizumab, carboplatin, pemetrexed | 10 (83.3) |
| Bevacizumab-awwb, carboplatin, pemetrexed | 1 (8.3) |
| Bevacizumab, gemcitabine | 1 (8.3) |
| **Line 2: Platinum-based chemotherapy combination^a^** | **33 (37.9)** |
| Carboplatin, pemetrexed | 14 (42.4) |
| Carboplatin, paclitaxel | 6 (18.2) |
| Carboplatin, paclitaxel protein-bound | 5 (15.2) |
| Carboplatin, gemcitabine | 5 (15.2) |
| Cisplatin, pemetrexed | 1 (3.0) |
| Carboplatin, docetaxel | 1 (3.0) |
| Cisplatin, gemcitabine | 1 (3.0) |
| **Line 2: Non-platinum-based chemo combination** | **2 (2.3)** |
| Paclitaxel protein-bound | 1 (50.0) |
| Gemcitabine, paclitaxel | 1 (50.0) |
| **Line 2: Single agent chemotherapy** | **11 (12.6)** |
| Pemetrexed | 6 (54.5) |
| Docetaxel | 3 (27.3) |
| Gemcitabine | 1 (9.1) |
| Paclitaxel | 1 (9.1) |
| **Line 2: Other therapy** | **1 (1.1)** |
| Ado-trastuzumab emtansine | 1 (100) |
| **Systemic Therapy Line 3** | **26 (11.4)** |
| **Line 3: Anti-PD-1/PD-L1-based therapy** | **8 (30.8)** |
| Pembrolizumab | 1 (12.5) |
| Nivolumab | 3 (37.5) |
| Carboplatin, pembrolizumab, pemetrexed | 2 (25.0) |
| Atezolizumab | 1 (12.5) |
| Cisplatin, pembrolizumab | 1 (12.5) |
| **Line 3: Anti-VEGF-based therapy** | **4 (15.4)** |
| Docetaxel, ramucirumab | 1 (25.0) |
| Bevacizumab, carboplatin, gemcitabine | 1 (25.0) |
| Bevacizumab-awwb | 1 (25.0) |
| Bevacizumab, carboplatin, paclitaxel protein-bound | 1 (25.0) |
| **Line 3: Platinum-based chemotherapy combination^a^** | **4 (15.4)** |
| Carboplatin, pemetrexed | 1 (25.0) |
| Carboplatin, paclitaxel | 2 (50.0) |
| Carboplatin, gemcitabine | 1 (25.0) |
| **Line 3: Single agent chemotherapy** | **9 (34.6)** |
| Docetaxel | 2 (22.2) |
| Gemcitabine | 3 (33.3) |
| Paclitaxel | 3 (33.3) |
| Vinorelbine | 1 (11.1) |
| **Line 3: Other therapy** | **1 (3.8)** |
| Afatinib | 1 (100) |
| **Systemic Therapy Line 4** | **7 (3.1)** |
| **Line 4: Anti-PD-1/PD-L1-based therapy** | **4 (57.1)** |
| Ipilimumab, nivolumab | 1 (25.0) |
| Atezolizumab | 1 (25.0) |
| Nivolumab | 1 (25.0) |
| Bevacizumab-awwb, pembrolizumab | 1 (25.0) |
| **Line 4: Platinum-based chemotherapy combination^a^** | **2 (28.6)** |
| Carboplatin, gemcitabine | 1 (50.0) |
| Carboplatin, paclitaxel | 1 (50.0) |
| **Line 4: Other therapy** | **1 (14.3)** |
| Crizotinib | 1 (100) |
| **Systemic Therapy Line 5** | **3 (1.3)** |
| **Line 5: Platinum-based chemotherapy combination^a^** | **2 (66.7)** |
| Carboplatin, paclitaxel | 1 (50.0) |
| Carboplatin, gemcitabine | 1 (50.0) |
| **Line 5: Other therapy** | **1 (33.3)** |
| Dabrafenib, trametinib | 1 (100) |

Data are n (%).

^a^ Platinum-based chemotherapy combinations without anti-VEGF agent.

PD1/PD-L1, programmed death 1/programmed death-ligand 1; VEGF, vascular endothelial growth factor.

**Supplementary Table 3 |** Reasons for first-line pembrolizumab discontinuation among 33 patients who received second-line and/or third-line anti-PD-1/PD-L1-based therapy: Spotlight cohort

|  | **Spotlight**  **(n = 33^a^)** |
| --- | --- |
| Recorded reason(s) for discontinuation, n (%) | *N=21^b^* |
| Progression | 8 (38.1) |
| Adverse effect of therapy | 6 (28.6) |
| Disease-related symptoms not due to therapy | 5 (23.8) |
| No evidence of disease | 2 (9.5) |
| Other^c^ | 1 (4.8) |

^a^ Three patients were treated with immune checkpoint inhibitor therapy in both second- and third-line.

^b^ Patients could have more than one reason for discontinuation. (The reason for discontinuation was missing for 12 patients.)

^c^ For patients with ongoing treatment until the time of death, the reason recorded was “Other” to comply with data deidentification requirements.

**Supplementary Table 4 |** Number of adverse events reported as related to pembrolizumab: Spotlight cohort.

|  | **Spotlight**  **(n = 228)** |
| --- | --- |
| Adverse events, n^a^ | 3 |
| Colitis | 1 |
| Cheilitis | 1 |
| Hypothyroidism | 1 |
| Serious adverse events, n | 0 |

^a^ Adverse events were identified during manual chart review, not actively solicited, and all were reported as related to pembrolizumab.
